# Association between the *ACCN1* Gene and Multiple Sclerosis in Central East Sardinia

**DOI:** 10.1371/journal.pone.0000480

**Published:** 2007-05-30

**Authors:** Luisa Bernardinelli, Salvatore Bruno Murgia, Pier Paolo Bitti, Luisa Foco, Raffaela Ferrai, Luigina Musu, Inga Prokopenko, Roberta Pastorino, Valeria Saddi, Anna Ticca, Maria Luisa Piras, David Roxbee Cox, Carlo Berzuini

**Affiliations:** 1 Dipartimento di Scienze Sanitarie Applicate e Psicocomportamentali, Università di Pavia, Pavia, Italy; 2 MRC Biostatistics Unit, Institute of Public Health, University Forvie Site, Cambridge, United Kingdom; 3 Divisione di Neurologia, Presidio Ospedaliero S. Francesco, ASL N°3 Nuoro, Nuoro, Italy; 4 Centro di Tipizzazione Tissutale, S.I.T., Presidio Ospedaliero S. Francesco, ASL N°3 Nuoro, Nuoro, Italy; 5 Nuffield College, Oxford, United Kingdom; 6 Department of Statistics, University of Oxford, Oxford, United Kingdom; 7 Dipartimento di Informatica e Sistemistica, Università di Pavia, Pavia, Italy; Baylor College of Medicine, United States of America

## Abstract

Multiple genome screens have been performed to identify regions in linkage or association with Multiple Sclerosis (MS, OMIM 126200), but little overlap has been found among them. This may be, in part, due to a low statistical power to detect small genetic effects and to genetic heterogeneity within and among the studied populations. Motivated by these considerations, we studied a very special population, namely that of Nuoro, Sardinia, Italy. This is an isolated, old, and genetically homogeneous population with high prevalence of MS. Our study sample includes both nuclear families and unrelated cases and controls. A multi-stage study design was adopted. In the first stage, microsatellites were typed in the 17q11.2 region, previously independently found to be in linkage with MS. One significant association was found at microsatellite D17S798. Next, a bioinformatic screening of the region surrounding this marker highlighted an interesting candidate MS susceptibility gene: the Amiloride-sensitive Cation Channel Neuronal 1 (*ACCN1*) gene. In the second stage of the study, we resequenced the exons and the 3′ untranslated (UTR) region of *ACCN1*, and investigated the MS association of Single Nucleotide Polymorphisms (SNPs) identified in that region. For this purpose, we developed a method of analysis where complete, phase-solved, posterior-weighted haplotype assignments are imputed for each study individual from incomplete, multi-locus, genotyping data. The imputed assignments provide an input to a number of proposed procedures for testing association at a microsatellite level or of a sequence of SNPs. These include a Mantel-Haenszel type test based on expected frequencies of pseudocase/pseudocontrol haplotypes, as well as permutation based tests, including a combination of permutation and weighted logistic regression analysis. Application of these methods allowed us to find a significant association between MS and the SNP rs28936 located in the 3′ UTR segment of *ACCN1* with *p* = 0.0004 (*p* = 0.002, after adjusting for multiple testing). This result is in tune with several recent experimental findings which suggest that *ACCN1* may play an important role in the pathogenesis of MS.

## Introduction

Multiple Sclerosis (MS) is a disabling disease of the central nervous system that affects young adults. Despite substantial evidence of polygenic inheritance of MS, the Major Histocompatibility Complex (MHC) is the only Deoxyribonucleic Acid (DNA) region where convincing evidence of linkage and association with MS has been found repeatedly and consistently [1], [2]. Multiple genome screens have nominated over 70 regions in linkage or associated with MS. Unfortunately, apart from some replications of linkage in regions of Chromosomes 5 and 17 [3], little overlap outside MHC has been found among these studies. Moreover, while each of the linkage studies has shown more allele sharing among affected individuals than would be expected by chance alone [4], none of them has succeeded in demonstrating linkage with MS at a genome-wide significance level. A possible explanation is the absence of MS susceptibility genes with strong individual effects. In addition, the statistical power to detect a modest effect may be low due to genetic heterogeneity within and among the studied populations. These considerations motivated our decision to study an isolated, genetically homogeneous, old population of the Italian province of Nuoro, Sardinia, where MS prevalence is 4–5 times as high as in the Italian mainland. The main objective of this paper is to report a genetic characterisation of MS in this very special population, and the discovery of an MS association with genes on Chromosome 17.

Our study is largely (but not exclusively) based on individuals extracted from families with multiple MS cases, where the genetic effect is expected to be stronger than in sporadic cases. Only a few DNA markers were genotyped and tested. This was made possible by adopting the following multi-stage design of the study. In the first stage, five microsatellites in the 17q11.2 region were typed and tested for MS association. One significant association was found at microsatellite D17S798. Next, a bioinformatic screen of the DNA region surrounding this microsatellite highlighted a strong candidate MS gene, called *ACCN1* (*Amiloride-sensitive Cation Channel Neuronal 1*, ENSG00000108684). Subsequent testing, exclusively of Single Nucleotide Polymorphisms (SNPs) located in *ACCN1*, revealed a significant association between MS and an SNP in the 3′ untranslated (UTR) region of that gene. This result is corroborated by several recent experimental findings, from independent sources, which we review in the Discussion section.

## Results

In 1998 we set up the current MS register in the province of Nuoro; for each MS case, the register provides the multigenerational pedigree. All diagnoses of MS were in accord with Poser's criteria for clinical definite MS [5]. An early analysis of the register data gave an estimated MS relative risk as high as 24 for a proband's sibling [6], and evidence that the risk of a proband's relative decreases with the degree of genetic sharing with the proband [7]. This suggests the presence of a strong genetic component of the disease in the studied population.

Our study sample consisted of a collection of small groups of individuals, which we call *nuclei*, each nucleus having been ascertained around an MS case, or *proband*, extracted from the above register. Most probands were extracted from a multiplex family, in consideration of the fact that cases with a strong family history of MS are more likely to exhibit an increased frequency of a particular susceptibility allele than sporadic cases [8]. We had 78 nuclei for a total of 229 individuals. The nuclei were subdivided according to type: 44 *type-1* nuclei consisting of a proband and of his/her parents, plus occasionally the proband's siblings; 22 *type-2* nuclei consisting of the proband, of his/her spouse and of their children, and 12 *type-3* nuclei consisting of a proband and of a corresponding unrelated control, matched by village of origin. Proband's siblings were present in five out of the 44 type-1 nuclei, with three nuclei including one sibling and the remaining two nuclei containing two. A large proportion (76.5%) of the 78 probands involved in our analysis was diagnosed during the relapsing-remitting stage of the MS course, and 38 of them were extracted from multiplex families with a varying number of affecteds.

Following the study design outlined in the Introduction, in the first stage of our analysis we separately tested for MS association five microsatellites under an MS linkage peak in the 17q11.2 region of DNA (for details see Genomic region and marker selection, data preparation and genotyping and Supplementary Table S1). In a previous meta analysis of three genome screens in the American, British and Canadian populations [9], this region had the highest linkage score for MS outside the MHC, with a non parametric logarithm of the odds (LOD) score of 2.58. Three types of association test, GLOBAL, LOCAL and regression-based (REG), are reported for each microsatellite in Table 1 (for an explanation of these tests see Statistical Analysis). Note that a consistently significant signal across the three tests is found only at microsatellite D17S798, with a *p*-value for the global null hypothesis of no association as low as 0.002, according to our REG test. The fact that the association signal found at D17S798 does not extend to the two microsatellites located about 2*cM* away from it is consistent with the fact that we found no evidence of linkage disequilibrium (LD) between the studied microsatellites. These findings may sound surprising when one considers that Zavattari *et al.* [10], in a work on Sardinian isolates published before the design of our study, estimated a 2*cM* persistence of LD between microsatellites. However, our findings are consistent with a very recent investigation on LD in Sardinia, notably by Service *et al.* [11], giving evidence that the extension of LD in old isolates, including the Nuoro population, is not as high as was expected.

**Table 1:** Association testing of each of the microsatellites selected for genotyping in the 17q11.2 region of DNA.

Microsatellite	Total number of alleles	GLOBAL^a^ *p-value*	LOCAL^b^ *minp*	REG^c^ *minp*
**D17s582**	6	0.90	0.90	0.69
**D17s1294**	6	0.86	0.98	0.80
**D17s1800**	8	0.11	0.03	0.09
**D17S798**	7	0.05	0.03	0.002
**D17s1850**	5	0.45	0.28	0.24

a *p*-value for the null hypothesis of no association of the microsatellite, calculated through the GLOBAL test procedure described in the Statistical analysis section.

b *p*-value for the null hypothesis of no association of the microsatellite, corresponding to the *minp*, π_r1_, obtained from the LOCAL testing procedure described in the Statistical analysis section.

c *p*-value for the null hypothesis of no association of the microsatellite, corresponding to the *minp*, π_r1_, obtained from the REG testing procedure described in the Statistical analysis section.

In our analysis of D17S798 we found that no alleles, except *1*, *5* and *6*, were sufficiently frequent in the studied population to contribute significant evidence of their association, given the available sample size. A comparison of the pseudocase *vs* pseudocontrol frequency for allele *5* (64 *vs* 43) and for allele *6* (52 *vs* 75) suggested that alleles *5* and *6* had a deleterious and protective effect, respectively, whereas allele 1 (31 *vs* 30) appeared to be neutral. Allele *6* of D17S798 had the most significant permutation-adjusted *p*-value according to both our REG test (π_r1_ = 0.002) and our LOCAL test (π_r1_ = 0.03). Next came allele *5*, with the second most significant permutation-adjusted *p*-value according to our REG test (π_r2_ = 0.003) and to our LOCAL test (π_r2_ = 0.034).

**Figure 1:**
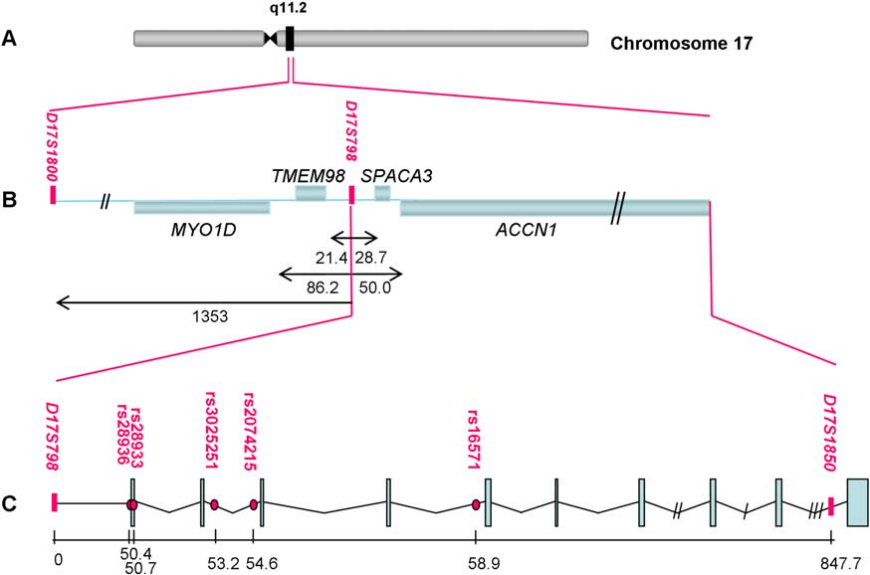
Studied genomic region. This figure consists of three panels, (A,B,C). Panel A shows the location of the 17q11.2 region on Chromosome 17. Panel B maps the position of four known genes (blue rectangles) in the region around D17S798, where a horizontal line represents DNA sequence, and a pink bar marks the location of microsatellites D17S798 and D17S1800. The diagram conveys the fact that TMEM98 and SPACA3 (represented above the horizontal line) lie on the forward helix, whereas Myo1D and ACCN1 (represented below the horizontal line) lie on the reverse helix. Panel C zooms on gene ACCN1, showing the locations of the genotyped markers relative to D17S798, in Kb. In this latter panel, the horizontal line represents genomic DNA, the pink dots represent the SNPs we have genotyped, the pink vertical bar represents microsatellite D17S1850, the blue bars represent ACCN1 exons and the wavy solid line between exons corresponds to the ACCN1 introns. The ACCN1 gene is located on the reverse strand, so the portion of ACCN1 near D17S798, where SNP rs28936 and SNP rs28933 are located, is the 3′ UTR-exon10 region of the gene. The width of the introns and exons in panel C is proportional to the actual length, with the exception of the first three very long introns (right portion of the panel) which have been shortened. Gaps are represented by diagonal bars: three bars (intron1-2) = 1,043,911 base pairs; two bars (intron 3–4) = 60,470 base pairs; one bar (intron 2–3) = 22,926 base pairs

Next we performed a bioinformatic screening of the DNA region surrounding D17S798, extending to the next studied microsatellite, on both sides. A map diagram of this region is shown in Figure 1. The only known genes in this region are *MYO1D* (*Myosin-Id*, ENSG00000176658) *TMEM98* (*Transmembrane protein 98,* ENSG00000006042), *SPACA3* (*Sperm Acrosome Associated 3*, ENSG00000141316) and *ACCN1* (the longest on chromosome 17). *MYO1D* is expressed in many tissues and codes a protein belonging to the family of unconventional myosins, playing its role in intracellular movements. *TMEM98* codes a small transmembrane protein of unknown function, while *SPACA3* codes an intra-acrosomal sperm protein mainly expressed in human spermatozoa and probably participating in the process of sperm-egg fusion during fertilization. Finally, *ACCN1* [12], [13] encodes the Mammalian Degenerin protein, a proton-gated channel permeable to sodium, lithium and potassium. The function of these channels is to generate ionic currents involved in the transmission of the nervous signal. While it cannot be excluded that *MYO1D*, *TMEM98* or *SPACA3* are involved in MS pathogenesis, *ACCN1* immediately appeared to be the only strong MS susceptibility candidate gene in the region explored (see the Discussion section for further bio-epidemiological evidence pointing at *ACCN1*).

Our subsequent analysis was entirely focused on the *ACCN1* gene, that extends from 50 Kb to 900 Kb away from D17S798. Direct sequencing of all the ten exons of this gene and their flanking regions (introns and UTR regions) highlighted 28 SNPs. These are described in Table 2. Of these, the five SNPs typed in bold in Table 2 were selected for testing, the selection criterion being based on minor allele frequency and quality control (for details see Genomic region and marker selection, data preparation and genotyping, and Tables S2 and S3). The last two columns of Table 3 report *p*-values for the null hypothesis of no association, for each of the five selected SNPs. These are permutation-adjusted in such a way to control for family-wise error rate under five-fold testing.

**Table 2:** Characteristics of the SNPs found on the *ACCN1* gene.

Internal code^d^	SNP rs number^e^	Alleles^f^	Physical distance (bp)^g^	Location in the gene	MAF^h^	Het (%)^i^	P HWE	Genotype frequency^j^			Call rate N (%)^k^
								wt/wt^l^	wt/snp	snp/snp	
1148110		*T/C*	28364120	3′ downstream	0.007	1.29	1	0.987	0.013	0	224 (95.32)
1147817		*A/G*	28364413	3′ UTR	0.007	1.29	0.0032	0.994	0	0.007	227 (96.60)
**1147727**	**rs28936** m	***A/G***	**28364503**	**3′ UTR**	**0.442**	**49.32**	**1**	**0.310**	**0.497**	**0.194**	**227 (96.60)**
1147715		*C/G*	28364515	3′ UTR	0.013	2.55	1	0.974	0.026	0	227 (96.60)
1147673	rs28935	*T/C*	28364557	3′ UTR	0.145	24.84	0.3130	0.716	0.277	0.007	216 (91.91)
1147653		*C/G*	28364577	3′ UTR	0.023	4.42	1	0.955	0.045	0	227 (96.60)
**1147435**	**rs28933**	***G/A***	**28364795**	**3′ UTR**	**0.427**	**48.94**	**1**	**0.329**	**0.487**	**0.184**	**232 (98.72)**
1147370	rs28932	*A/G*	28364860	3′ UTR	0.052	9.84	0.0503	0.909	0.078	0.013	226 (96.17)
1147289		*C/T*	28364941	3′ UTR	0.007	1.41	1	0.986	0.014	0	208 (88.51)
1147285		*G/A*	28364945	3′ UTR	0.004	0.70	1	0.993	0.007	0	209 (88.94)
1147090		*C/T*	28365140	Exon 10	0.004	0.70	1	0.993	0.007	0	208 (88.51)
1145331		*C/T*	28366899	Intron 9–10	0.004	0.78	1	0.992	0.008	0	186 (79.15)
**1144942**	**rs3025251**	***G/A***	**28367288**	**Intron 8–9**	**0.199**	**31.92**	**0.4456**	**0.628**	**0.346**	**0.026**	**221 (94.04)**
1144881		*A/G*	28367349	Intron 8–9	0	0	n.a.	1.000	0	0	179 (76.17)
1143866		*A/G*	28368364	Intron 8–9	0.020	3.84	1	0.961	0.039	0	224 (95.32)
**1143568**	**rs2074215**	***G/A***	**28368662**	**Intron 8–9**	**0.332**	**44.37**	**1**	**0.112**	**0.441**	**0.447**	**221 (94.04)**
1143255		*G/A*	28368975	Intron 7–8	0.017	3.28	1	0.967	0.033	0	219 (93.19)
1143254		*C/T*	28368976	Intron 7–8	0.056	10.50	0.0648	0.902	0.085	0.013	225 (95.74)
1143085		*C/T*	28369145	Intron 7–8	0.009	1.74	1	0.982	0.018	0	157 (66.81)
1139477		*C/T*	28372753	Intron 6–7	0.004	0.70	1	0.993	0.007	0	204 (86.81)
1139258	rs11868226	*T/A*	28372972	Intron 6–7	0.019	3.77	1	0.962	0.039	0	186 (79.15)
**1139226**	**rs16571**	***C/T***	**28373004**	**Intron 6–7**	**0.447**	**49.45**	**0.5121**	**0.290**	**0.526**	**0.184**	**221 (94.04)**
1136996	rs16569	*C/T*	28375234	Intron 5–6	0	0	n.a.	0	0	1	222 (94.47)
1135185	rs16967902	*G/A*	28377045	Intron 5–6	0.026	5.08	1	0.948	0.052	0	224 (95.32)
1132904		*G/A*	28379326	Intron 4–5	0.007	1.31	1	0.987	0.013	0	223 (94.89)
1132889		*C/-*	28379341	Intron 4–5	0	0.00	n.a.	0	0	1	223 (94.89)
1049212	rs9916605	*G/A*	28463018	Intron 2–3	0.076	13.99	0.039	0.868	0.112	0.020	222 (94.47)
1048984		*C/G*	28463246	Intron 1–2	0	0	n.a.	1	0	0	225 (95.74)

d Code assigned by CNG.

e Rs numbers retrieved from the HapMap Project [25].

f Allele on the left side of the slash is the wild type allele, allele on the right side of the slash is the minor frequency allele.

g Physical distances are referred to NCBI Human Genome Build 36.

h MAF = Minor Allele Frequency calculated on 160 founders.

i Heterozygosity - calculated on 160 founders as genotype frequencies.

j Calculated on the whole sample.

k Number of individuals (percentage) of samples with non-missing data over the total number of genotyped samples.

l “wt” indicates the major allele and “snp” the minor allele.

m SNPs analyzed are in bold.

Out of the five SNPs tested, only rs28936 and rs28933 appear to be significantly associated with MS, the former being significantly associated under both the LOCAL and the REG test. These two SNPs are closer to D17S798 than any of the remaining three SNPs we have tested, and very close to each other. rs28936 and rs28933 were also in full LD with each other (D′ = 1, R^2^ = 0.91, see Tables S4 and S5). In Table 3, the *p*-values obtained by using our REG test (for details see Statistical analysis: regression based tests of association section) are consistently lower than those obtained by using the LOCAL test. This may be due to a greater sensitivity of the REG test, owing to the fact that it incorporates the uncertainty due to the imputation of haplotypes. By means of logistic regression we also estimated the effect size for SNP rs28936, corresponding to an odds ratio of 2.07 (1.4–3.1 95% CI) for the *A* allele of this SNP, under a multiplicative model. In a conditional analysis performed by using weighted regression (for details see Statistical analysis: regression based tests of association section), when we conditioned on the most significant SNP, rs28936, no residual association was provided by the remaining four SNPs, suggesting no evidence of multiple effects operating across the studied SNPs. However, after conditioning on SNP rs28936, residual association was still detected at the D17S798 microsatellite (*p*-value = 0.03), suggesting that another effect might operate within *ACCN1*, or in a different gene, or in a non-coding region in LD with D17S798. Note that our tests for association tended to be conservative, since they were based on haplotypes imputed under the null hypothesis of no association.

**Table 3:** Association testing of each of the five SNPs genotyped in the *ACCN1*.

SNP	Variant allele	Pseudo cases^n^ *(exp freq)*	Pseudo controls^o^ *(exp freq)*	LOCAL^p^ *p-value*	REG^q^ *p-value*
**rs28936**	*G*	57.0	83.0	0.0078 (0.02)	0.0004 (0.002)
**rs28933**	*A*	55.8	78.4	0.017 (N.S.)	0.002 (0.01)
**rs3025251**	*A*	27.6	16.6	0.204 (N.S.)	0.135 (N.S.)
**rs2074215**	*A*	99.3	112.0	0.116 (N.S.)	0.062 (N.S.)
**rs16571**	*T*	72.1	69	0.727 (N.S.)	0.676 (N.S.)

n Expected frequency of pseudocases carrying the variant allele.

o Expected frequency of pseudocontrols carrying the variant allele.

p The first number is the unadjusted *p*-value for the null hypothesis of no association of the SNP, based on the Z statistic for the 2×2 table comparing the two alleles of the SNP. The second number, in brackets, is the corresponding adjusted version of the *p*-value, calculated through the LOCAL test procedure described in the Statistical analysis section, to correct for the five-fold multiplicity.

q The first number is the unadjusted *p*-value for the null hypothesis of no association of the SNP, based on an unconditional weighted logistic regression. The second number, in brackets, is the corresponding adjusted version of the *p*-value, calculated through the REG test procedure described in the Statistical analysis section, to correct for the five-fold multiplicity. The symbol “N.S.” stands for “statistically non significant”.

Finally, we tested for disease association the haplotype formed by D17S798 and SNP rs28936. Based on both our LOCAL and our REG test (giving *minp* values of 0.044 and 0.025, respectively) we could reject the null hypothesis that no variant of that haplotype was associated with disease. The “*5.A*” haplotypic variant, representing a combination of allele *5* of D17S798 and the “*A*” allele of SNP rs28936, had 43.8 expected pseudo-cases *vs* 22.5 expected pseudo-controls, which suggested a deleterious effect of this variant. By contrast the “*6.G*” variant, with 22.1 expected pseudocases *vs* 46.5 expected pseudocontrols, appeared to have a protective effect. The remaining variants (“*1.A*”, “*1.G*”, “*5.G*”, “*6.A*”) appeared to have a neutral effect. Indeed, the variants “*6.G*” and “*5.A*” had the most significant and second most significant associations, respectively, with a *p*-value (π_r1_) for variant “*6.G*” as low as 0.027 in our REG test (and π_r1_ = 0.044 in our LOCAL test), and a *p*-value (π_r2_) for variant “*5.A*” as low as 0.025 in our REG test (and π_r2_ = 0.069 in our LOCAL test). The adjusted *p*-values for the “*6.G*” and “*5.A*” variants were considerably larger than the *p*-value for SNP rs28936 alone.

## Discussion

We have studied MS association in the genomic region 17q11.2 in the isolated population of Nuoro, Sardinia. In the first stage of our study we analysed five microsatellites scattered across an independently discovered MS linkage peak in 17q11.2. We found one significant MS association at microsatellite D17S798. Of the known genes surrounding this microsatellite, *ACCN1* was identified to be the best MS susceptibility candidate on the basis of external, *a priori*, information. This prompted us to resequence the exons and the 3′ UTR segment of *ACCN1*, where SNPs were identified and tested for MS association. Two SNPs (rs28936 and rs28933) in the 3′ UTR region of *ACCN1* gave significant marginal associations after adjusting for five-fold SNP testing. Confidence in a genuine association is strengthened by the fact that the two most significant associations were found on two SNPs that occupy adjacent positions in the sequence of tested markers, and, moreover, by the fact that both these significant SNPs have a high frequency of the minor allele. Moreover, consider that the effect size for the deleterious allele of SNP rs28936, on an odds ratio scale, was as high as 2.07 (1.4–3.1 95% CI). Odds ratios of this magnitude are not unexpected in a genetically homogeneous, high disease prevalence, population. The power to detect such an effect by our method, given our sample size, is greater than 87% for any value of α greater than 0.01. Recall that our method, due to the particular permutation procedure, may lose sensitivity in those situations where the conditioning on nucleus founders is unnecessary. When we conditioned on SNP rs28936, no residual association was provided by the remaining four SNPs, suggesting no evidence of multiple effects operating across the studied SNPs. In the following we discuss *(i)* current knowledge about the biology of *ACCN1*, and *(ii)* external evidence supporting an involvement of *ACCN1* in MS. Finally we shall suggest a possible role of *ACCN1* in the aetiology of MS, in the light of the available evidence.

The *ACCN1* gene, a member of the family of amiloride sensitive cation channel acid sensing ion channels [13], encodes the Mammalian Degenerin (MDEG, Q16515) protein, a proton-gated channel permeable to sodium, lithium and potassium. The function of these channels is to generate ionic currents involved in the transmission of the nervous signal. MDEG has two isoforms, MDEG1 and MDEG2, with different biological properties [14]. MDEG1, expressed in the postsynaptic membrane of granule cells and in the Purkinje cells of the cerebellum, can form an active ionic channel either as a standalone, or by binding other proteins of its family, and is activated by a low pH. MDEG2, expressed in the brain and in sensory neurons, cannot form an active ionic channel as a standalone, but participates in an heterodimeric active ionic channel by interacting with another protein of its family. For example, MDEG2 interacts with the *Amiloride-sensitive Cation Channel 3* (*ACCN3*) to form a channel where the ionic current is modulated by the Protein Kinase C, presumably the alpha isoform, [15] via shared interaction with the Protein Kinase C Alpha Binding Protein (PRKCABP), an adaptor protein encoded by the Protein Interacting with C Kinase 1 (*PICK1*, ENSG00000100151) gene. Interestingly, an heteromer formed by the above proteins is involved in the sensation of pain caused by low pH values. Furthermore, there is evidence that MDEG participates in mechanosensation, perception of taste, perception of pain and possibly in neurotransmission and neuromodulation [16]. In addition, mice knock-out experiments have shown that it is required for normal light-touch sensation [17]. See [16] for a review of current understanding of the *ACCN1* gene and its family, including their potential pathogenetic role and the possibility of therapeutic modification. The hypothesis of an involvement of *ACCN1* in MS is well within the realms of possibility, as indicated by the following experimental, clinical and epidemiological evidence:

1. In the nematode *Caenorhabditis elegans*, mutations of MDEG homologues known as degenerins (deg-1, mec-4, mec-10) are the major known causes of hereditary neurodegeneration, according to experimental literature [12].
2. The MDEG1 channel is constitutively activated in the presence of the same mutation that causes degeneration in the nematode [12]. This has led to the suggestion that a gain of function of the MDEG1 channel might be involved in human forms of neurodegeneration.
3. A link between *ACCN1* and neurodegeneration, suggested in the above two points, appears particularly interesting in the light of recent evidence that the primary insult in MS may indeed be of a neurodegenerative (rather than inflammatory) kind [18]. Such a kind of insult, it is suggested in [18], might bring in its wake a harmful autoimmunity, itself causing further collateral damage to neurons, in the context of a positive feedback link that might well set the pace of a progressive neuronal injury at certain stages of MS or in some MS patients.
4. An involvement of a deregulated ion channel in MS has been reported [19]. More specifically cerebellar ataxia, a sign that accompanies MS, seems to be due to an increased expression of sensory-neuron-specific sodium channels in the Purkinje cells, leading to increased ionic current inside the cells and to consequent alterations in cellular functions.

The following points also indicate, albeit rather indirectly, plausibility of an involvement of *ACCN1* in MS:

- there is now evidence that susceptibility to common autoimmune disorders such as Type 1 diabetes, Graves' disease and autoimmune hypothyroidism [20], [21] may largely depend on genetic variation in untranslated genic regions, possibly involving a modification of the localization, stability or splicing of the messenger Ribonucleic Acids (mRNAs). This suggests that a deregulated expression of *ACCN1*, possibly an overexpression resulting in an increased ionic current when the channel is activated, may underlie our finding of an MS association at the rs28936 polymorphism in the 3′ UTR region of *ACCN1*;
- an association between the *Protein Kinase C alpha* (*PRKCA*, ENSG00000154229) and MS has been observed in the Finnish and Canadian populations [22]. In the light of the previously discussed functional link between the Protein Kinase C alpha and MDEG2 (encoded by the *ACCN1* gene), the above finding provides indirect prior evidence in favour of a genuine involvement of *ACCN1* in MS patho-genesis, thereby adding weight to the results of our analysis. These findings, combined with the results our study, point to a possible interaction between PRKCA and *ACCN1* in the context of MS association, a possible target for future investigation;
- the region 17q11.2 where *ACCN1* gene is located is syntenic to the *Eae18b* QTL on rat Chromosome 10. This locus of approximately 3Mb in length was found to be associated with the murine model of multiple sclerosis EAE (Experimental Autoimmune Encephalomyelitis), displaying maximum LOD scores in the range 4.5 to 5.8, depending on the EAE phenotype. The *ACCN1* gene is included in this linkage interval and the peak linkage marker D10Rat123 is internal to the gene [23].

We would add that secondary inflammation in MS, as long as it is accompanied by acidosis, might activate the MDEG1 ion channel, and consequently lead to cell death in the context of a progressive, inflammation-mediated, neurodegenerative process. Our results point to a family of ion channels which are considered to be potential drug targets because they are involved in neuropathic pain. If the above hypotheses are true, future drugs for neuropathic pain might work also in the treatment of MS, perhaps as a prophylactic measure to prevent inflammation in the early stage of the disease, or in combination with immunosuppressor drugs in the subsequent stages of the disease. In our opinion, the above considerations motivate further experimental and epidemiological investigation of the role of *ACCN1* in MS aetiology. Finally, our statistical results do not exclude presence of other SNPs within (or in the proximity of) *ACCN1*, that are also associated with MS, thereby motivating future, more extensive, exploration of the genomic region in the vicinity of D17S798.

## Materials and Methods

### Genomic region and marker selection, data preparation and genotyping

Table S1 describes the five high heterozygosity microsatellites which we selected for typing in 17q11.2. Some were used in previously mentioned genome screens, or located near markers used in previously published work.

All the ten exons of *ACCN1* and their flanking regions (introns and UTRs) were directly sequenced, which led to identifying 28 SNPs. All of these were found in non-coding regions, with the exception of the SNP 1147090 found in exon number 10. This non-synonymous SNP leads to a Pro499Ser substitution; however, we excluded it from the association analysis due to the very low minor allele frequency. Of the 28 identified SNPs, the five SNPs highlighted in bold in Table 2 were selected for inclusion in the association analysis. The criteria for selection were: *(i)* a minimum call rate of 90%, *(ii)* consistency with Hardy-Weinberg Equilibrium (HWE) in founders at a *p*-value ≥0.01 *(iii)* presence of homozygous genotypes of major/minor alleles and presence of the heterozygous genotype, *(iv)* heterozygosity greater than 0.25, *(v)* a minor allele frequency (MAF) greater than 10%. The choice of 10% as a threshold for MAF was motivated by the fact that, given our sample size, an SNP with an MAF below 10% cannot contribute appreciable evidence of an association.

From the schematic diagram of Figure 1 it appears that two of the five selected SNPs are located in the 3′ UTR region flanking exon 10, two are located within intron 8–9, and one is located in intron 6–7. Because *ACCN1* is a very long gene, with as many as ten exons and with the longest intron (intron 1–2) in its chromosome [24], our chosen SNPs do not provide a complete coverage of it, with the exception that the 3′ UTR portion of the gene was found to contain all the tag SNPs listed in the HapMap Project (see http:// www.hapmap.org [25]) for that portion of the gene. Moreover, we improved the coverage by including in the analysis the microsatellite D17S1850, located within the gene in the intron 1–2. Exon sequencing details are given in Tables S2 and S3. The blood sample collection was performed at the Division of Neurology, S.Francesco Hospital, Nuoro (I); the buffy coat preparation and the DNA extraction, according to classical salting out protocol, were performed at the Centro di Tipizzazione Tissutale of the Azienda Sanitaria Locale N°3, Nuoro, where the biological bank resides. Further DNA extraction from buffy coat, microsatellite typing and gene sequencing were performed at the Centre National de Genotypage, Evry, France (see http://www.cng.fr/).

### Statistical analysis

In a classical trio study, an ascertained proband is genotyped along with his or her parents to form a *proband-parent trio*. The present study extends this design by including two further ascertainment schemes. In the first, a proband is genotyped along with his or her spouse (and possibly their offspring). In the second, an isolated proband is recruited into the study to act as an isolated case with a corresponding control, matched by village of origin. Our study embraces all these three ascertainment schemes, by including proband-parent trios, which we call *type-1 nuclei*, proband-spouse-offspring trios, which we call *type-2 nuclei*, and matched case-control pairs, which we call *type-3 nuclei*. A *founder* is any individual who has no parents included in his/her own nucleus. Thus, in a nucleus of type 1 or 2 the two parents are the founders, whereas in a nucleus of type 3, where there is no offspring, both the proband and the corresponding matched control are founders.

### Pseudocases and Pseudocontrols

We regard a family-based association study as a special case of matched case/control analysis, where each nucleus contributes *pseudocases* and *pseudocontrols*. Biernacka and Cordell [26] define pseudocases and pseudocontrols at a genotype level. We favour the following, different, option: in type-1 nuclei, we define the pseudocases to be the two parental haplotypes transmitted to the proband, and the pseudocontrols to be the remaining two parental haplotypes. In type-2 nuclei, we define the pseudocases to be the two haplotypes found in the proband, and the pseudocontrols to be those found in the spouse. In type-3 nuclei, we define the pseudo-cases to be the two haplotypes found in the proband, and the pseudocontrols to be those found in the proband's matched control. Under a null hypothesis of no disease association at the studied loci, any haplotype variant should be approximately equally frequent in the pseudocases and in the pseudocontrols, under each of the three different ascertainment schemes. We pool the three types of nuclei into a joint analysis which looks for haplotype variants that are significantly more (or less) frequent in the pseudo-cases than in the pseudocontrols. A large disparity between the pseudocase and pseudocontrol counts, for any particular haplotypic variant, may be taken to indicate evidence of an association. In the spirit of the Transmission Disequilibrium Test [27], our analysis acknowledges the mutual matching of pseudocases and pseudocontrols in the same nucleus.

One reason for choosing a haplotype-level analysis, rather than a genotype-level one, is that in the presence of occasional genotyping failures the missing allele calls can be imputed by “borrowing strength” from information about the neighbouring loci, so as to exploit linkage disequilibrium over the studied DNA region. Given a set of genotyping data, at multiple loci, we estimated the (imperfectly observed) haplotypic phase and, at the same time, imputed any missing calls at a particular locus by combining information about population-level haplotypic frequencies with information about the neighbouring loci. The ability to “borrow strength” from information about neighbouring loci will attenuate the impact of biased missingness, for example when an SNP is preferentially losing heterozygotes. The method avoids the need to eliminate families with ambiguous haplotypes, which would incur bias, and moreover allows tests of association to be performed at a haplotype level, whenever appropriate [28], [29].

### Reconstructing the Haplotypes

Our data include incomplete genotype measurements in the studied founders, denoted by GF, incomplete genotype measurements in the offspring, denoted by GO, and the ascertainment status of each studied individual, represented by a vector D pointing at individuals with an ascertainment proband status. Unknown quantities in the problem are complete haplotype information for all founders, HF, and complete haplotype information for all offspring, HO. We use data (GF,GO, D) to make inferences about (HF,HO), under the assumption that *(i)* no recombinations within the studied DNA region occur in the parent-to-offspring meioses, and that *(ii)* genotyping error is absent. Violations of the latter assumption were preliminarily checked by looking at locus-specific deviations from HWE, and at mendelian consistency between parental and offspring genotypes. Consider the Bayesian posterior for HF, denoted as π(HF) and proportional to P(HF,GF,GO,D). Because under a null assumption of no disease association D is independent of (HF,GF,GO), we may write:
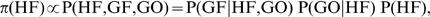
where ∝ stands for “proportional to”. Because GF is a deterministic function of HF, and therefore independent of GO, once we know HF, we may simplify this as follows:
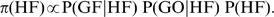
Under assumption *(ii)* above, P(GF|HF) indicates logical consistency between the founder haplotype assignments, HF, and the corresponding observed genotypes, GF. The term P(GO|HF), in type-1 and type-2 nuclei, reflects mendelian consistency between founder haplotypes and observed offspring genotypes (depending on the proportion of patterns of transmission of the parental haplotypes which match the observed offspring genotypes). The term P(HF) represents prior assumptions about the population distribution of haplotypes (see below).

We use the MCMC-based PHASE software [30] to draw from π(HF) samples of HF, denoted HF^(1)^, HF^(2)^, …, HF^(M)^. In this software, P(HF) is shaped around the population genetics model proposed by Stephens et al [31]. Next, in order to identify the pseudocases and the pseudocontrols in each nucleus, we associate each HF^(i)^, (for *i* = 1,…,*M*), with a corresponding sample of HO, denoted by HO^(i)^, extracted from P(HO| HF^(i)^,GO). Note that P(HO|HF,GO) specifies in each nucleus the probability of any particular haplotype assignment in the offspring, conditional on the founders' haplotypes and on the available genotyping data. We end up with a set {(HF^(i)^,HO^(i)^)} of samples whose empirical distribution approximates the posterior distribution of (HF,HO). From these samples, we obtain the probability of any particular pseudocase/control assignment in any particular nucleus, which enables us to generate a *reconstruction table*. This is a table where each row corresponds to a distinct, posterior-weighted, pseudocase/control assignment in a particular nucleus. Each nucleus generally contributes separate rows in the reconstruction table, one row for each distinct pseudocase/control assignment with positive probability in the posterior, for that nucleus. Shown in Table 4 is a pedagogic example of a reconstruction table with only three nuclei represented in it. In this example, the first row represents an assignment where founder 1 of nucleus 264 has haplotypes *AGT* and *AAC*, both acting as pseudocases, whereas the third row represents an assignment where founder 1 of nucleus 1121 has case haplotype *GGT* and control haplotype *AAC*. In our analysis, each pseudocase or pseudocontrol haplotype in the table was defined over the complete sequence of DNA loci under study. In the last column of the table, a (posterior) weight gives the relative proportion of times that any particular haplotype assignment in a particular nucleus was generated by the Markov chain. Recall that the reconstruction table has been generated under a null hypothesis of no association. Any test of association based on such a table, including the tests we are going to propose, will tend to be conservative, as a consequence of this fact.

**Table 4:** Pedagogic example of a reconstruction table with only three nuclei represented in it. See explanation in the main text.

Family	Nucleus type		Founder 1		Founder 2		Weight
			Pseudocase	Pseudocontrol	Pseudocase	Pseudocontrol	
264	2	assignment 1	*AGT*			*GGT*	0.35
			*AAC*			*GGT*	
264	2	assignment 2	*GGT*			*GGT*	0.65
			*AAC*			*AGT*	
1121	1	assignment 1	*GGT*	*AAC*	*GAC*	*GGC*	0.25
1121	1	assignment 2	*GAT*	*AGC*	*GAC*	*GGC*	0.50
1121	1	assignment 3	*GGT*	*AAC*	*GGC*	*GAC*	0.25
660	1	assignment 1	*GGT*	*AAC*	*GGC*	*GGC*	1.0

### A GLOBAL Test of Microsatellite Association

The *p*-values in column 3 of Table 1 have been obtained by separately applying to each of a set of five microsatellites the following GLOBAL test of association. Let our global null hypothesis, H_0_, assert that none of the *m* alleles of a microsatellite is associated with the disease. To reject/accept H_0_ as a whole, we proceed as follows. From the reconstruction table we obtain, by weighted averaging, the expected frequency of copies of allele *j* that are pseudocases (*t*_ij_) and pseudocontrols (*u*_ij_) in nucleus *i*, for all relevant combinations of *i* and *j*. By summing over the nuclei we obtain *d*_j_ = Σ_i_(t_ij_−*u*_ij_), and *d*  = (*d*_1_, …, *d*_m_). A marked departure of *d*_j_ from zero indicates a deleterious (or protective) effect of allelic variant *j* on disease risk. Under H_0_ we can freely interchange the entire vector *t*_i_ = (*t*_i1_, …, *t*_im_) with *u*_i_ = (*u*_i1_, …, *u*_im_), with probability 0.5, independently in each *i*-th nucleus. Under the resulting permutation distribution, *d* has zero expectation and variance covariance matrix *V*: {*v*_hk_ = Σ*_i_*(*t_ih_*−*u* *_ih_*)(*t_ik_*−*u* *_ik_*)}. A global test of H_0_ can be based on the fact that under such permutation distribution the statistic *Z* = *d*'*V* ^−^ *d* (where *V* ^−^ denotes the generalized inverse of *V*) has an asymptotic 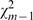 distribution. Because the test is performed under a null permutation distribution which conditions on the set of founder haplotypes in each nucleus, it is robust to population stratification differences *between* nuclei. Due to the presence of type-3 nuclei, the method is not robust to population stratification differences *within* the nucleus founders. The problem is attenuated by the fact that we study an isolated population, and that we match the two founders of each type-3 nucleus by village of origin.

### LOCAL Tests via Permutation

The above, global, test may be undersensitive when departure from the null hypothesis is entirely explained by one or few alleles of a highly polymorphic microsatellite. We may then consider the following, alternative, LOCAL test procedure. Each allele is compared against the group of all others by forming a 2×2 table of pseudocase/control expected frequencies, generated by weighted averaging over the reconstruction table. Let *T* be the largest of the *Z* statistics of 2×2 tables, each of which compares one allele against all others. To obtain an empirical *p*-value for H_0_, denoted *minp*, we compare *T* with its distribution under random permutations of the reconstruction table. These are performed by randomly interchanging the pseudocase with the pseudocontrol haplotypes, with probability 0.5 independently in each row of the reconstruction table. The same properties of robustness we have mentioned in the context of our GLOBAL test hold for this LOCAL test. It should be noted that, when we omit the pseudocase/control labels of the haplotypes, the reconstruction table does not provide information about the null hypothesis of no disease association, yet it is representative of the uncertainty about nuisance parameters related to the unobserved haplotypic phase and missing allele calls. By testing association via permutations of the pseudocase/control labels, independently in each row of the reconstruction table, we come close to conditioning on the values of a complex set of nuisance parameters unrelated to the null hypothesis.

A comparison between columns 3 and 4 of Table 1 shows a clear tendency of the *minp* calculated through the LOCAL test to be more sensitive than the test of the previous section, in the study of our microsatellites. A similar procedure we have used to test association of the five SNPs of the *ACCN1* gene. First, we have calculated a separate unadjusted *p*-value for each SNP, essentially by treating it as a bi-morphic microsatellite so that the test of the previous section could be applied. We have then ordered the unadjusted *p*-values for the various SNPs such that *p*_r1_≤*p*_r2_≤…≤*p*_rm_. In order to adjust for multiple SNP testing, each *p*_rj_ is then transformed into a corresponding adjusted *p*-value, π_rj_, using the permuted replicates of the reconstruction table we had previously generated in the analysis of microsatellites. From each of these replicates, an ordered set of *p*-values for the various SNPs, *P*_r1_≤*P*_r2_≤…≤*P*_rm_, is obtained. Essentially, we calculate π_rj_ as the proportion of replicates where *p*_rj_ is equal to or less than *min*(*P*_rj_, …≤*P*_rm_), with a possible modification required to ensure monotonicity of the {π_rj_} [32], [33]. The procedure ensures a strong control of the family-wise error rate under multiple SNP testing. Column 5 of Table 3 contains *p*-values calculated through the procedure just described. Because our permutation procedure preserves correlation between SNPs, the reported *p*-values acknowledge non-independence of the tests.

### Regression-Based Tests of Association

The test procedures described so far are based on expected pseudocase/control frequencies, and therefore ignore uncertainty due to haplotype reconstruction. An alternative, inspired by a paper by Cordell [34], is to use the following REG test based on unconditional logistic regression [35], [36]. With reference to our analysis of the five SNPs of Table 3, the idea is to perform a separate regression analysis on each individual SNP. In the regression, each of the four haplotypes in each row of the reconstruction table is treated as an independently observed response/covariate pair, where the response is 0 for a pseudocontrol and 1 for a pseudocase, and where the covariate represents presence/absence of the minor allele in the SNP of interest. By opting for the “weighted logistic regression” option found in most statistical packages, we allow the observations contributed by any particular row of the reconstruction table to enter the regression with a relative importance fixed by the corresponding posterior weight. For each *j*th SNP, we obtain a corresponding estimate of the relative risk parameter, βj, and a corresponding Wald's statistic and *p*-value. The *p*-values (one for each SNP) are then permutation-adjusted to account for multiple SNP testing. Embedding Cordell's regression approach within a permutation scheme protects from the possibility that the weighting scheme used in the regression does not rigorously correspond to a likelihood in a specific model of the data. Because of its ability to incorporate haplotype assignment uncertainty, the REG test should be more sensitive than the LOCAL test, and the results of Table 3 seem to confirm this. We also used an extension of the above REG procedure, where a permutation-based *p*-value for the hypothesis of no association of a *target* marker is computed by adjusting for the effect of a *conditioning* marker. This simply requires including a covariate term for *both* the conditioning and target markers in the regression equation.

The data management and the statistical analysis of the reconstructed haplotype configurations, according to the proposed methods, were performed with the aid of the software package GADA (Genetic Association Downstream Analysis), written in R by one author (CB).

## Supporting Information

Table S1 Characteristics of the analyzed microsatellites.
(0.06 MB DOC)

Table S2 Primer names and nucleotidic sequence used for *ACCN1* exon resequencing.
(0.05 MB DOC)

Table S3 Primer names, PCR conditions and sequence conditions for the exons of the *ACCN1* gene.
(0.05 MB DOC)

Table S4 R^2^ coefficient for each pair of studied SNPs (information from the founders of the Nuoro population and from the Caucasian population).
(0.05 MB DOC)

Table S5 D′ coefficient for each pair of studied SNPs (information from the founders of the Nuoro population and from the Caucasian population).
(0.05 MB DOC)

Table S6 Minor allele frequency of studied SNPs (information from the founders of the Nuoro population and from the Caucasian population).
(0.05 MB DOC)
