# Supplementary material for: Association between the ACCN1 Gene and Multiple Sclerosis in Central East Sardinia
Source: PLoS One. 2007 May 30;2(5):e480. doi: 10.1371/journal.pone.0000480 (PMC1868958; doi:10.1371/journal.pone.0000480)
Supplement: Table S1 — Characteristics of the analyzed microsatellites. (0.06 MB DOC) [file pone.0000480.s001.doc]

**Table S1. Characteristics of the analyzed microsatellites.**

| Microsatellite  within 17q11.2 | Genetic distance (cM) | Physical distance (bp) | Het (%) | *p*-value HWE | Call rate (%) |
| --- | --- | --- | --- | --- | --- |
| D17S582 | ≈ 50.74 | 23135259 | 68.22 | 0.51 | 90.21 |
| D17S1294**b,f** | 50.74 | 25406301 | 75.71 | 0.05 | 95.32 |
| D17S1800**a** | 51.63 | 26960942 | 79.53 | 0.49 | 95.32 |
| D17S798**abc** | 53.41 | 28314091 | 67.66 | 0.04 | 99.57 |
| D17S1850 | 55.33 | 29161843 | 53.71 | 0.38 | 97.02 |

Table S1 describes the five microsatellites selected in the 17q11.2 genomic region for typing and analysis. Lower case literals within microsatellite label indicate: ***(a)*** microsatellite chosen on the basis of evidence of MS linkage in American screen **[1], *(b)*** microsatellite chosen on the basis of evidence of MS linkage in British screen **[2],** ***(c)*** microsatellite chosen on the basis of evidence of MS linkage in Canadian screen **[3]** and ***(f)*** microsatellite chosen on the basis of evidence of MS linkage in Finnish screen **[4].**

Genetic distances were taken from the Marshfield map (<http://research.marshfieldclinic.org/genetics/MarkerSearch/buildMap.asp>). Physical distances are based on the NCBI Human Genome Build 36 (<http://www.ncbi.nlm.nih.gov/Genome>)**.** The heterozygosity values reported in the “Het” column are calculated according to **[5]**. Call rate is calculated as the percentage of samples with non-missing data at the specific locus over the total number of genotyped samples. The column headed “*p*-value HWE” reports the *p*-value for the test for departure from Hardy-Weinberg equilibrium (HWE), based on the nucleus founders. A *p*-value smaller than 0.05 for D17S798 indicates that this microsatellite significantly departs from HWE, this particular deviation being due to an excess of homozygotes for the protective allele of the marker. Such a deviation is an expected consequence of the protective effect of the allele. In such a situation, parental allele frequencies do not reflect general population frequencies, in those trios that have been ascertained on the basis of an affected proband. In an analysis of the probands, we found no evidence of a significant deviation from HWE. This result is consistent with the presence of a genuine disease association under a multiplicative model **[6,7]**. In addition, consider that *(i)* almost no mendelian inconsistencies were detected in a genotype-oriented, single locus, check of our family data, based on the PEDCHECK program **[8]**, and *(ii)* each sample was genotyped twice at *D17S798*, using different primers, and substantial concordance between the two allele calls was found. We also note that the particular kind of deviation from HWE, corresponding to an excess of homozygotes for the protective allele in parents, will, in the kind of analysis we performed, lead to a more conservative result. The above considerations suggest that the deviation from HWE detected in the founders at microsatellite D17S798 is unlikely to be due to genotyping error.

Whenever, in a trio, a locus was found to be “inconsistent” according to PEDCHECK, the genotype of each trio member at that locus was treated as “missing”. We also checked the pedigree structures with the aid of the program IBSCheck developed by Simon Heath, based on information at a specified set of microsatellites. Hardy-Weinberg Equilibrium (HWE) at microsatellites was tested by using the function gtab developed as part of David Clayton’s GENASSOC package (<http://www-gene.cimr.cam.ac.uk/clayton/software/stata/genassoc/>) programmed in STATA (<http://www.stata.com/>). Hardy-Weinberg Equilibrium at each SNP was tested by using the function HWSNP written by M.A. Cleves and available at <http://biostat-resources.com/stata>.

1. Haines JL, Ter-Minassian M, Bazyk A, Gusella JF, Kim DJ, et al. (1996) A complete genomic screen for multiple sclerosis underscores a role for the major histocompatability complex. Nat Genet 13: 469-471.

2. Sawcer S, Jones HB, Feakes R, Gray J, Smaldon N, et al. (1996) A genome screen in multiple sclerosis reveals susceptibility loci on chromosome 6p21 and 17q22. Nat Genet 13: 464-468**.**

3. Ebers GC, Kukay K, Bulman DE, Sadovnick AD, Rice G, et al. (1996) A full genome search in multiple sclerosis. Nat Genet 13: 472-476.

4. Kuokkanen S, Gschwend M, Rioux JD, Daly MJ, Terwilliger JD, et al. (1997) Genomewide scan of multiple sclerosis in Finnish multiplex families. Am J of Hum Genet 61: 1379-1387.

5. Ott J (1992) Strategies for characterizing highly polymorphic markers in human gene mapping Am J of Hum Genet 51: 283.-290.

6. Nielsen DM, Ehm MG, Weir BS (1998) Detecting marker-disease association by testing for Hardy-Weinberg disequilibrium at a marker locus. Am J of Hum Genet 63: 1531-1540.

7. Wittke-Thompson JK, Pluzhnikov A, Cox NJ (2005) Rational inferences about departures from Hardy-Weinberg equilibrium. Am J of Hum Genet 76:967-968.

8. O'Connell JR, Weeks DE (1998) PedCheck: a program for identification of genotype incompatibilities in linkage analysis. Am J of Hum Genet 63: 259-266.
