# Supplementary material for: Association between the ACCN1 Gene and Multiple Sclerosis in Central East Sardinia
Source: PLoS One. 2007 May 30;2(5):e480. doi: 10.1371/journal.pone.0000480 (PMC1868958; doi:10.1371/journal.pone.0000480)
Supplement: Table S2 — Primer names and nucleotidic sequence used for ACCN1 exon resequencing. (0.05 MB DOC) [file pone.0000480.s002.doc]

**Table S2. Primer names and nucleotidic sequence used for *ACCN1* exon resequencing.**

| **EXON** | **PRIMER NAME** | **PRIMER** |
| --- | --- | --- |
| EXON 1 | ACCN1_EX1_PF | CATCACCTTGGTGTCTCCCT |
| EXON 1 | ACCN1_EX1_PR | AGGCACTGCTCTCTCTCCTG |
| EXON 1 | NP-ACCN1_EX1_PF2 | TGTAACCTGAATGGCTTCCG |
| EXON 2 | ACCN1_EX2_PF | GCTGTCAGATTTCTGGGGTC |
| EXON 2 | ACCN1_EX2_PR | CCAAGCCAGGGACATCTTTA |
| EXON 3 | ACCN1_EX3_PF | CAATCACACCTGCAATGTCC |
| EXON 3 | ACCN1_EX3_PR | CAGTAAAGCCCTTGACCGAG |
| EXON 4 | NP-ACCN1_EX4_PF | GGAGCCAGATGTTGAAGGTC |
| EXON 4 | NP-ACCN1_EX4_PR | AAGTTTCCACCTGCCCTCTT |
| EXON 4 | NP-ACCN1_EX4_SF | TCCTGTCTTGCAGCTCACAT |
| EXON 4 | NP-ACCN1_EX4_SR | GCGAAGTGGGTGAAGTGTTT |
| EXON 5 | NP-ACCN1_EX5_PF | AGCCTGCCCAAGCTTACATA |
| EXON 5 | NP-ACCN1_EX5_PR | GCTCCTCTGATGCCTACCTG |
| EXON 5 | NP-ACCN1_EX5_SF | CAGAGGAGGTGTTGCTGACA |
| EXON 5 | NP-ACCN1_EX5_SR | CCCTTCCCAACCTTCTCTTC |
| EXON 6 | 50007DH04_PF | GCAGGGAAGAAAGCCATGAG |
| EXON 6 | 50007DH04_PR | CCTCCAATTTCCTCCTTATCCAG |
| EXON 7 | 00064BC01_PF | TCTGCTCCTCTCTTCGCCAG |
| EXON 7 | 00064BC01_PR | AAGACCATGAATTCCAAGAGTGC |
| EXON 7 | 00064BC01_SF | GGCAATTGACTTGATAATGCCAG |
| EXON 7 | 00064BC01_SR | TGCTTTCTCTTTGCACCCTATG |
| EXON 8 | 00064BD01_PF | CACCTGTCACCAGTCCACAC |
| EXON 8 | 00064BD01_PR | TGGAAGTTCTGGAGCCATTG |
| EXON 8 | 00064BD01_SF | TGGGTTGCATCTCAGTCTGTTC |
| EXON 8 | 00064BD01_SR | CGCCTTCCCTCTACTATGATTCTG |
| EXON 9 | 00064BE01_PF | TGTTCATGACCAAAGGCTGC |
| EXON 9 | 00064BE01_PR | CTGAGCCCAGGAGTAGACTGAG |
| EXON 9 | 00064BE01_SF | TGGGCTTGTGCTACGGTATG |
| EXON 9 | 00064BE01_SR | ATGGAAAGGTGTGCAGTGAGTC |
| EXON 10 | 00114EH08_PF | TCAAACCAGGGAGCCTTCTC |
| EXON 10 | 00114EH08_PR | AGGGCAGCCTCCTTCAGAAC |
| EXON 10 | 00114EH08_SF | AACGAACCTGCCATCACATC |
| EXON 10 | 00114EH08_SR | TTGGGCAGGTTAAGAACTTTCAC |
