# Supplementary material for: Association between the ACCN1 Gene and Multiple Sclerosis in Central East Sardinia
Source: PLoS One. 2007 May 30;2(5):e480. doi: 10.1371/journal.pone.0000480 (PMC1868958; doi:10.1371/journal.pone.0000480)
Supplement: Table S3 — Primer names, PCR conditions and sequence conditions for the exons of the ACCN1 gene. (0.05 MB DOC) [file pone.0000480.s003.doc]

**Table S3. Primer names, PCR conditions and sequence conditions for *ACCN1* exons**

| EXON | PRIMER name | PCR*  CONDITIONS | | | PRIMERS PCR | SEQUENCE  CONDITIONS | | PRIMERS SEQUENCE |
| --- | --- | --- | --- | --- | --- | --- | --- | --- |
|  |  | C˚ | CYCLE | Taq polymerases |  | C˚ | CYCLE |  |
| EXON 1(1st part) | ACCN1_EX1 | 55 | 35 | hercu | PF,PR | 60 | 25 | PF |
| EXON 1(2nd part) | NP_ACCN1_EX1_PF2 | 55 | 35 | hercu | PF,PR | 60 | 25 | PF2 |
| EXON 2 | ACCN1_EX2 | 60 | 35 | taq | PF,R | 60 | 25 | F,PR |
| EXON 3 | ACCN1_EX3 | 58 | 35 | hercu | PF,PR | 60 | 25 | PF,PR |
| EXON 4 | NP-ACCN1_EX4 | 60 | 35 | hercu | SF,PR | 60 | 25 | SF,SR |
| EXON 5 | NP-ACCN1_EX5 | 60 | 35 | taq | PF,PR | 60 | 25 | SF,SR |
| EXON 6 | 5007DH04 | 60 | 35 | taq | PF,PR | 60 | 25 | PF,PR |
| EXON 7 | 64BC01 | 60 | 35 | taq | PF,PR | 60 | 25 | SF,SR |
| EXON 8 | 64BD01 | 60 | 35 | taq | PF,PR | 60 | 25 | SF,SR |
| EXON 9 | 64BE01 | 62 | 35 | taq | PF,PR | 60 | 25 | SF,SR |
| EXON 10 | 114EH08 | 62 | 35 | taq | PF,PR | 60 | 25 | SF,SR,PR |

* Two Taq polymerases were used depending on the fragment. Hercu: Herculase from Stratagene taq: Ex Taq from Takara Bio Inc. (Kyoto Japan).
