# Supplementary material for: Association between the ACCN1 Gene and Multiple Sclerosis in Central East Sardinia
Source: PLoS One. 2007 May 30;2(5):e480. doi: 10.1371/journal.pone.0000480 (PMC1868958; doi:10.1371/journal.pone.0000480)
Supplement: Table S4 — R2 coefficient for each pair of studied SNPs (information from the founders of the Nuoro population and from the Caucasian population). (0.05 MB DOC) [file pone.0000480.s004.doc]

**Table S4. R^2^ coefficient for each pair of studied SNPs (information from the founders of the Nuoro population and from the Caucasian population).**

|  | **rs28936** | **rs28933** | **rs3025251** | **rs2074215** |
| --- | --- | --- | --- | --- |
| **rs28933** | 0.91[[1]](#footnote-2)  1[[2]](#footnote-3) |  |  |  |
| **rs3025251** | 0.31  -[[3]](#footnote-4) | 0.34  - |  |  |
| **rs2074215** | 0.39  0.46 | 0.36  0.46 | 0.12  - |  |
| **rs16571** | 0.01  - | 0.00  - | 0.30  - | 0.40  - |

1. Upper number is the R^2^ in the Nuoro population. [↑](#footnote-ref-2)
2. Lower number is the R^2^ in the Caucasian population (data from the HapMap Project). [↑](#footnote-ref-3)
3. The symbol”-“ stands for “unavailable”. [↑](#footnote-ref-4)
