# Supplementary material for: Association between the ACCN1 Gene and Multiple Sclerosis in Central East Sardinia
Source: PLoS One. 2007 May 30;2(5):e480. doi: 10.1371/journal.pone.0000480 (PMC1868958; doi:10.1371/journal.pone.0000480)
Supplement: Table S5 — D′ coefficient for each pair of studied SNPs (information from the founders of the Nuoro population and from the Caucasian population). (0.05 MB DOC) [file pone.0000480.s005.doc]

**Table S5. D’ coefficient for each pair of studied SNPs (information from the founders of the Nuoro population and from the Caucasian population).**

|  | **rs28936** | **rs28933** | **rs3025251** | **rs2074215** |
| --- | --- | --- | --- | --- |
| **rs28933** | 1.00[[1]](#footnote-2)  1[[2]](#footnote-3) |  |  |  |
| **rs3025251** | 1.00  -[[3]](#footnote-4) | 1.00  - |  |  |
| **rs2074215** | 1.00  1 | 1.00  1 | 1.00  - |  |
| **rs16571** | 0.08  - | 0.03  - | 1.00  - | 1.00  - |

1. Upper number is the D’ in the Nuoro population. [↑](#footnote-ref-2)
2. Lower number is the D’ in the Caucasian population (data from the HapMap Project). [↑](#footnote-ref-3)
3. The symbol”-“ stands for “unavailable”. [↑](#footnote-ref-4)
