# Supplementary material for: Association between the ACCN1 Gene and Multiple Sclerosis in Central East Sardinia
Source: PLoS One. 2007 May 30;2(5):e480. doi: 10.1371/journal.pone.0000480 (PMC1868958; doi:10.1371/journal.pone.0000480)
Supplement: Table S6 — Minor allele frequency of studied SNPs (information from the founders of the Nuoro population and from the Caucasian population). (0.05 MB DOC) [file pone.0000480.s006.doc]

**Table S6. Minor allele frequency of studied SNPs (information from the founders of the Nuoro population and from the Caucasian population).**

|  | **SNP**  **Minor Allele Frequency** % | | | | |
| --- | --- | --- | --- | --- | --- |
| Population | **rs28936** | **rs28933** | **rs3025251** | **rs2074215** | **rs16571** |
| Nuoro | 44  **(A)[[1]](#footnote-2)** | 50  **(G/A)** | 11  **(A)** | 28  **(G)** | 44  **(T)** |
| **Caucasian[[2]](#footnote-3)** | 32  (G) | 32  (A) | - | 49  (A) | - |

1. Minor allele in brackets. Frequencies were calculated in the Nuoro pseudocontrols and in the Caucasian population. [↑](#footnote-ref-2)
2. Data from the HapMap Project. [↑](#footnote-ref-3)
